# AI Ethics beyond Principles: Strengthening the Life-world Perspective

**DOI:** 10.1007/s11948-025-00530-7

**Published:** 2025-02-10

**Authors:** Stefan Heuser, Jochen Steil, Sabine Salloch

**Affiliations:** 1 Institute for Protestant Theology and Religious Education, Technische Universität Braunschweig, Braunschweig, Germany; 2 Institute for Robotics and Process Control, Technische Universität Braunschweig, Braunschweig, Germany; 3 Hannover Medical School, Institute for Ethics, History and Philosophy of Medicine, Hannover, Germany

**Keywords:** Artificial intelligence, Health care, Ethics, Ethical theory, Ethical principles

## Abstract

The search for ethical guidance in the development of artificial intelligence (AI) systems, especially in healthcare and decision support, remains a crucial effort. So far, principles usually serve as the main reference points to achieve ethically correct implementations. Based on reviewing classical criticism of principle-based ethics and taking into account the severity and potentially life-changing relevance of decisions assisted by AI-driven systems, we argue for strengthening a complementary perspective that focuses on the life-world as ensembles of practices which shape people’s lives. This perspective focuses on the notion of ethical judgment sensitive to life forms, arguing that principles alone do not guarantee ethicality in a moral world that is rather a joint construction of reality than a matter of mere control. We conclude that it is essential to support and supplement the implementation of moral principles in the development of AI systems for decision-making in healthcare by recognizing the normative relevance of life forms and practices in ethical judgment.

## Background: Human-AI Interaction in Healthcare

Novel forms of interaction between humans and artificial intelligence (AI)-driven systems in various domains of healthcare (such as the diagnostics of rare diseases, chatbots for psychotherapy, or clinical decision support systems) increasingly affect practices, expertise and, indeed, the ethos of healthcare professionals, as well as the lives of patients and their relatives. AI-systems are not just tools or machines that serve to achieve human purposes but technical media that contribute to the modelling of purposes and the courses of action (Hubig, 2021). What is more, they may even be attributed or simply take on the role of intelligent, contingent counterparts in interaction with humans (Nassehi, 2024). As is the case with medical diagnosis systems, deep learning convolutional neural networks are able to make judgments or decisions that may compete or even outperform human expertise (Liu et al., 2019).

Against this background, the ethics of AI in healthcare encompasses highly important problems that lie on a spectrum between rather obvious sources of moral danger and extremely hidden and intricate issues that can only be identified with a high level of technical expertise together with a sufficient degree of ethical sensitivity. Attempts to systematize ethical issues that arise in medical AI have been made distinguishing between (a) epistemic issues, related to misguided, inconclusive or inscrutable evidence, (b) normative issues, related to unfair outcomes and transformative effects (e.g. racism and discrimination), and (c) issues of traceability (Morley et al., 2020). In addition, ethical quandaries that need to be solved occur at all stages, from early technological development up to AI implementation and surveillance, in healthcare practice. The micro level of direct interaction between patients and healthcare providers needs to be considered as well as the institutional (“meso”) perspective and the level of healthcare systems.

There is obviously a need for (ethical and legal) guidance on AI in healthcare to ensure that the quality of care is not infringed against the background of technological progress and increased efficiency. Political initiatives on international and national levels as well as innumerable ethics councils, boards and initiatives have presented results of comprehensive and usually interdisciplinary work. Prominent examples include the European high-level expert group on AI and the German Ethics Council which focus on the regulation of the development and use of AI systems by means of law, guidelines and moral principles (Deutscher Ethikrat, 2023; European Commission, 2019).

The need for appropriate and effective normative guidance challenges moral enquiry to find new answers to old questions: How far can moral principles cover the normatively relevant phenomena of human life– and at which point do we need additional approaches to the field of morality?

While affirming the value of principle-based approaches, we will argue in this article that ethical reflection on the development of medical AI needs to explicitly concern those aspects of normativity which cannot be sufficiently captured within a system of principles. On the one hand, our approach touches upon the question how principles of AI ethics can be operationalized in AI development (Artificial Intelligence Ethics Impact Group, 2020). On the other hand, the interaction between humans and AI systems, which ranges from assisted or automated decision-making to aid in the activities of daily living, calls for a expanded perception of the forms and facts of human life and the tasks that come with it. Applications of machine learning in the healthcare and assistance sectors obviously relate to issues of privacy, autonomy, vulnerability and fairness. At the same time, the complex embeddedness of AI applications in social, organizational and normative contexts suggests that a mere juxtaposition of frameworks of moral principles versus cultures of human life forms will not suffice to meet the normatively relevant challenges.

These considerations lead us to the following set of questions: How can moral principles and aspects of “the good life” in the diversity of its forms and practices help to understand, evaluate and inform the interaction between human beings and intelligent systems? How can normatively relevant dimensions of the life-world and its practices be brought to bear on AI development in an ethically responsible way? How far can the human capacity of judgment help to align the functional imperatives of AI systems with the complex grammar of the human life-world, with its diversity of practices and tasks?

This article sets out with a brief overview of four domains of critique of ethical principles as guiding norms in the evaluation of medical AI. It then draws on the idea of human life forms and highlights the role of moral judgment which cannot be circumvented as a basis for developing alternative models for the ethical guidance of AI in healthcare. Finally, the article discusses some concrete implications for ethical guidance and practice. Our arguments in general aim to contribute to the interdisciplinary discourse on appropriate types of ethical guidance for medical AI. The results are relevant for experts, bodies and institutions that issue ethics guidelines for AI in healthcare. Likewise, the consequences of our approach bear on the conceptual frameworks of ethics on which the development of medical AI systems is based and also touch upon ethical issues of the clinical use of AI.

## Ethical Principles as Guiding Norms in the Evaluation of Medical AI

Approaches to guiding the ethical assessment of AI in healthcare so far often refer to ethical *principles* as normative precepts that are supposed to facilitate ethically legitimate answers to concrete moral problems. A vast number of (profit and non-profit) organizations and ethical boards have issued principle-based ethical guidelines in recent years. Prominent examples are– among others– the opinions of the European High Level Group on Ethics (European Commission, 2019) and the French and German national councils on ethics (Comité National Pilote D’Éthique du Numérique, 2022; Deutscher Ethikrat, 2023). Five ethical principles (beneficence, non-maleficence, autonomy, justice and explicability) for AI development and adoption were prominently introduced by the AI4People initiative in 2018, based on an assessment of 20 AI-related recommendations (Floridi et al., 2018). A review on the “global landscape” of AI ethics guidelines was published by Jobin and colleagues (2019) leading to the conclusion that guideline efforts need to be accompanied by ethical analysis and adequate implementation strategies.

Vast critique, however, has been voiced in recent years towards taking principle-based accounts as the guiding rationale for AI ethics (Munn, 2023). This article also analyses the shortcomings inherent to principle-based accounts regarding the ethical evaluation of AI in healthcare by distinguishing four different levels of argument. Problems mainly arise with respect to:

- ethical theory,
- the canon of ethical principles,
- social characteristics of AI development and implementation, and
- the need to find regulations.

We will briefly depict these four domains and tentatively approach additional elements that are missing to provide adequate AI guidance.

### Ethical Theory

The focus on ethical principles as guiding rationales in dealing with the normative insecurity emerging from increasingly powerful AI technologies is per se not surprising against the background of bioethics having been a predominantly “principle-driven” discipline in the past few decades. Since its first publication in 1979, Beauchamp and Childress’ *Principles of Biomedical Ethics* has very much dominated the emergence of the new discipline in its many academic and applied facets. It also led to profound debates on this type of principlism being a theoretical foundation for resolving bioethical problems. Even if the academic dispute between principlism, casuistry and deductivism that took place in the early 1990s is directed predominantly to the four-principles approach, the main features of the debate are still insightful for the theoretical foundation of AI ethics today.

It has been argued from the side of the “theorists” that the four principles are lacking any systematic relationship, which makes them inappropriate for solving practical conflicts which often consist of a contradiction between two or more of the principles. According to Clouser and Gert (1990), the four principles “are in fact not guides to action, but rather they are merely names for a collection of sometimes superficially related matters for consideration when dealing with a moral problem”. They criticized that without the embedding in an overarching ethical theory, the principles could not help for clear and coherent decision procedures. In practice, the danger was seen that autonomy, beneficence, non-maleficence and justice would be applied only superficially (e.g. by doctors), leading to an overestimation of the validity of ethical decision-making. More concretely, Holm (1995) argued that methods of specification and balancing, as described by Beauchamp and Childress (1979), could lead to a preponderance of autonomy at the expense of the other principles in evaluating concrete cases.

On the other hand, critique was voiced from authors who argue from a casuist tradition and consider the four principles as an abstract theoretical guidance that cannot fruitfully be linked to concrete situations, for example, in clinical practice. As argued for by Arras (1994), our concrete moral stances do not derive from theoretical standpoints, but emerge from the evidence of exemplary cases with which we are familiar. If moral conflicts arise, a reference to abstract principles does not help, but the answer needs to be read out of the concrete circumstances of each situation. Not completely contrary to this view, other authors defend Beauchamp and Childress’ (1979) approach in arguing that with an appropriate understanding of the underlying weak moral intuitionism, the four-principles approach might be well-qualified for dealing with concrete bioethical cases (Quante & Vieth, 2002).

What can we learn from these more or less historic debates for the current challenges ascribed to principle-based accounts towards the ethics of AI? The tension between ethical theory and (clinical, research or policy) practice still occurs in a similar form (Bleher & Braun, 2023), generating the need to explain how a finite list of principles can guide concrete decision-making. Whereas the need for an overarching ethical theory might be stressed from the philosophical side, the role of the perceptions and the moral knowledge of clinicians, developers, patients and other stakeholders needs to be integrated. This knowledge has a “casuist” character as it relies on the practical dealing with concrete cases. As we could see from the debate on “principlism”, even if “theorists” and “casuists” take very different starting points, they agree in their analyses that a further factor– be it a faculty or a procedure– is needed to make the judgment on concrete ethical issues reliable and prevent it from becoming biased or misguided.

### The Canon of Principles

When discussing the ethics of medical AI, we naturally work with the intersection of at least two branches of ethical analysis: AI ethics and medical ethics. If we decide to broaden the perspective even more, domains of applied ethics need to be involved, such as ethics of technology (or robot ethics, in particular) and nursing ethics. Depending on one’s viewpoint on applied ethics, the segmentation of ethical approaches with distinct theoretical frameworks appears as helpful or not. The fragmentation of applied ethics into different specialized fields, each having its own normative framework, can be criticized. The well-known plurality of normative theories in general philosophical ethics often does not lead to major practical problems. It even serves as a reference point for fruitful discussions about ethical concepts and the nature of morality, and leads to an intensive reflection about the interrelation between different types of theory. This situation changes drastically as soon as we move to applied ethics and its dealing with concrete problems that do not have a scenario or experimental character but immediately affect humans and other entities (Salloch, 2016). Here, the selection of an ethical framework clearly matters with respect to the concrete outcomes. How far an applied ethics theory needs to mirror the contextual characteristics of its field of application is a meta-ethical point that has not yet been fully agreed on (Zichy et al., 2012).

Regarding the ethical assessment of medical AI, Jobin and colleagues (2019) demonstrated that the ethical principles of transparency, justice and fairness, non-maleficence and responsibility play a dominant role in the existing guidelines. By contrast, the category of “freedom and autonomy” was quantitatively far less important, which stands in stark contrast to biomedical ethics, with its strong focus on patient’s self-determination. Thus, the question arises, which set of ethical principles is about to dominate the development and implementation of medical AI, for example, whether medical AI ethics is approached more from the point of view of AI ethics or biomedical ethics. Even more fundamentally, whether the idea of a distinct set of principles “fitting” its subject should be supported at all needs to be scrutinized. Such preconditions of principle-guided approaches to AI ethics have not yet been sufficiently clarified. Dealing with them requires a competent analysis and meta-reflection on the character and limitations of ethical principles as guiding rationales.

### Social Characteristics of AI Development and Implementation

Ethics codes specifically directed to AI developers have so far often been issued by industry stakeholders (Bitkom, 2024); huge tech companies especially present themselves at the forefront of promoting such guidance (Google, 2018). “Hippocratic Oaths” for developers are frequently called for in the academic and public spheres, but effective guidance is often missing. Existing codes of conduct and professional ethics guides (IEEE, 2020) that are available in engineering at large and specifically for computer science (Association for Computing Machinery, 2018), as well as best practices and norms for software design (ISO, 2019), are often not followed. Reasons for this might be linked to a lack of education of the developers and high economic and competitive pressure on the market. Empirical research hints to the fact that this deficit might also be associated with a missing recognition of their own ethical agency by AI developers (Griffin et al., 2023). Historical, social and institutional factors, however, also need to be taken into account.

It has become apparent in current debates on the ethics of medical AI that the long tradition of dealing with principle-based ethical approaches in clinical practice and biomedical research cannot be easily transferred to the domain of AI development and implementation. This is because the social practice of AI development differs significantly from healthcare. Mittelstadt (2019) describes these differences trenchantly in pointing to five characteristics that distinguish AI development from clinical medicine. At first, he argues that AI developers do not share common aims and adhere to fiduciary duties in the same way as healthcare professionals. As AI development is not a formal profession, it does not operate within the same regulatory frameworks, and practices might differ considerably. Secondly, AI development cannot draw on the same professional history and norms that are still powerful in healthcare practice and lead to a “homogeneous professional culture and identity” (Mittelstadt, 2019). Furthermore healthcare has available various methods and structures to translate high-level ethical principles into practice such as “[p]rofessional societies and boards, ethics review committees, accreditation and licensing schemes, peer self-governance, codes of conduct, and other mechanisms” (Mittelstadt, 2019). Whereas AI development also shows first attempts at establishing ethics implementation structures, it cannot draw on the same repertoire as healthcare does so far. Finally, Mittelstadt (2019) argues that legal and professional accountability is often less clear in AI development than in the various domains of healthcare.

Such historical, social and institutional factors shed further light on what needs to be considered from a pragmatic perspective when referring to ethical principles as guiding rationales for medical AI. We cannot expect that they are just easily incorporated into concrete decision-making, but their adherence is dependent on various aspects that come along with historical and contingent factors of medical or AI practice, respectively.

### Need for Regulation

A fourth domain of potential shortcomings of principle-based accounts to AI evaluation relates to the need for concrete regulation and guidance. The idea of single or team decision makers balancing principles for the treatment of specific cases does not seem sufficient as a depiction of the real landscape of the introduction of AI into healthcare. Besides to the individual level, institutional (meso) and political (macro) structures are extremely powerful for the concrete effects of AI being used. Trustworthiness, for example, as highlighted by the High-level Expert Group on AI (European Commission, 2019), does not relate mainly to individual actors but to structures that need to be established on the institutional and political levels. From a philosophical perspective, the same holds true for autonomy as a principle less often referred to in the AI debate (Jobin et al., 2019) but of utmost importance in healthcare. Autonomy understood as purely individualistic has come under attack from various sides as it could “encourage ethically questionable forms of individualism and self-expression” (O’Neill, 2002, p. 73). If we think about appropriate ethical frameworks for AI ethics, the individual level of decision-making needs to be considered in its interwovenness with institutional and regulatory aspects that might be more powerful in AI guidance in the conducive as well as the misleading perspective.

As a consequence, the addressee of ethical principles included in AI ethics guidelines and statements is far from being obvious. Instead, a comprehensive reflection is necessary on which ethical demands might be sensibly addressed at which level and by whom. How the micro (e.g. patient-physician decision-making), meso (healthcare institutions) and macro level (e.g. health policy and politics) interrelate and continuously influence each other also needs to be considered.

## The Importance of Ethical Judgment for AI Ethics in Healthcare

As demonstrated above, the collaborative search for adequate ethical guidance, for means and measures of its implementation in research, product development and in the interaction between humans and intelligent systems remains of paramount importance for the alignment of AI systems, particularly in the field of healthcare. Protection against racism and discrimination, the violation of rights as well as issues of privacy and of data protection cannot be imposed on the individual user and should not be left only to technological solutions, but also need to be addressed politically. However, despite all efforts to place the development and use of AI systems within the frameworks of moral principles and law, crucial questions in AI ethics remain unresolved. As we will show in the following section, these questions are closely linked to human “life forms” and the way they are involved in the interaction between humans and AI systems.

### Human Life Forms: A Challenge for AI Ethics

In our understanding, the term “life form” refers to historically and culturally variable but inert “ensembles of social practices” (Jaeggi, 2018, p. 33), into which people have grown, which configure the ways in which they live the basic processes of human life (such as pregnancy, being born, growing up, raising children, being ill and recovering, caring, aging or dying), and which guide their social interactions. According to Jaeggi “forms of life are nexuses of practices, orientations, and orders of social behavior. They include attitudes and habitualized modes of conduct with a *normative character* that concern the *collective conduct of life*, although at the same time they are *not strictly codified* or institutionally binding” (2018, p. 50 [emphasis in original]). Understood in this way, life forms are not just habits– although habitualized behaviour is part of them– but practical figurations which people would describe as part of their unquestioned conceptions of a “good” life.

Although social philosophy and sociology have long taken an interest in the impact of the introduction of AI systems on cultures, social spheres and concepts of reality (Floridi, 2014), the normative implications of the question of how interactions of humans and AI systems can meaningfully respond to, continue and even transform or extend human life forms still need to be explored. While a lot of the architecture and operations of AI systems function very well even as “black boxes”, the contents of human life forms cannot be skipped without emptying ethical judgment from the rich moral texture of culturally embedded practices, trajectories, imaginations, intuitions, experiences, tacit knowledge, habits, skills, hopes and emotions. These forms and practices of a lived life can be normatively relevant and, hence, need to be taken into account by those who issue ethics guidelines for AI in healthcare and by medical AI systems developers in order to facilitate adequate technological responses to human life practices– but also to reveal which aspects of these practices cannot be translated into systemic interaction. In order to find such *good*, not only morally and legally *correct* ways of human-machine-interaction, AI ethics is, therefore, challenged to focus both on the moral principles and legal norms that guide the interaction between humans and AI systems *and* on specific life forms, practices and language games that are touched upon by such interaction.

In this quest, we may draw on findings of the classical debate in moral philosophy on the distinction between questions of justice and the good life, and on the mutual dependence of these questions (Rawls, 1991; Sandel, 1984). According to this debate, universal moral principles concerning all human beings and requiring their reasonable consent need to be distinguished from but, at the same time, are closely related to the life forms and practices through which people find, participate in and contribute to what they may call a good life (Eisikovits & Feldman, 2022). The results of this debate can be applied to the interaction between human beings and intelligent systems, which also calls for a distinct but closely related use of principle- and life form-based approaches to ethics. While the concrete character of the relationship between these two approaches to ethics has remained controversial in this classical debate, we propose that this requires an extended concept of ethical judgment that is able to combine normatively relevant aspects of the life-world with moral principles. Merely technomorphic or moralistic viewpoints on the interaction of humans and machines will not suffice. We also need to critically investigate the ways in which ethical judgment helps to discern both the world of humans and machines and the moral principles that guide their interaction.

### The Discerning Capacity of Ethical Judgments

Which aspects of human life forms are normatively relevant for conceptualizing and guiding the interaction of humans with intelligent systems? And how can these aspects be brought to bear within this interaction– alongside the orientation conveyed by moral principles and legal rules? We strive to find answers to these questions by adding judgments which are sensitive to life forms to the concept of ethical reasoning. And because these should be implemented in the models and practical procedures of the interaction of humans and AI systems, the respective answers are fundamental for guiding the development of such systems and consequently to develop guidelines for such development.

An important point of reference for such a concept of ethical judgment sensitive to life forms is still Immanuel Kant’s critique of judgment, in which the competence of judgment does not only entail cognitive but also sensory aspects (1913). While a lot of the discourse on AI ethics has concentrated on the conditions of human-machine interaction to be determined by moral principles (sometimes drawing on Kant’s critique of practical reason) (Kim & Schönecker, 2022), Kant’s critique of judgment helps to extend the capacity of judgment towards an interplay of aesthetic and moral elements, thereby remaining in close contact to and yet distinct from the reality that affects the judging subject. As Hannah Arendt has shown in her Kant lectures (1982), it is by this power of judgment that people constitute a common world which they can inhabit and experience together. Judgment should, hence, not be reduced to the moral orientation of action by means of principles, but also has an explorative task directed towards the constitutive forms and practices that form our common world (Wegner, 2023). It is this competence to discern through which people come to articulate their place in the world with others in the life forms that they share, and through which they find out how to continue their life with others according to these shared life forms. Judgments, thus, imply the question whether people follow the paths that are already laid out in their shared life forms– or whether they deviate from these paths. *Ethical* judgment, in this sense, becomes virulent in situations where decisions have to be made about how to continue living in the face of cuts into the regular course of life, as can be the case in severe accidents or diseases. It is, therefore, helpful, if not necessary, to use this capacity of judgment in situations of (shared) decision-making in healthcare settings and to apply it to the interaction with AI-driven decision support. Even if many examples of medical AI appear as non-critical applications that guide risky and potentially far-reaching decisions in clinical practice, we need to consider the impact on the shared life forms of those stakeholders who are affected.

### Ethical Judgment in Healthcare: The Case of AI-based Decision-making

The application of AI-based *decision support systems* seems particularly promising in data-heavy fields of healthcare (Rajkomar et al., 2019) and has invoked a discussion on questions of trust and distrust, medical errors, clinical outcomes, deskilling and the role of professional experience (Gaube et al., 2021). While AI machines are highly capable of processing and providing information and analysis, they lack an embodied, practical relationship to the world as a common place to live one’s life in all its forms and practices. This emphasizes the importance of the introduction of discerning judgments to the interaction of humans and AI systems, specifically when it comes to the shaping of decision-making processes. The same applies, of course, to decision-making in other fields of the human life-world, in which decisions need to be both morally compliant and placed within shared life forms, contexts, stories and histories of beings who coexist, and who have to live with the consequences of their decisions.

AI-based decision support systems are a specific example for a general phenomenon in healthcare decision-making. Be it assisted by machines or not, decision-making never takes place in a vacuum. It is, instead, both related to the world of moral principles *and* embedded in a complex life-world of diversified life forms. Ethical judgment in decision-making, thus, needs to identify the paths that are traced by the forms of life of the people concerned, and find ways of pursuing these paths, of altering them or to seek new ones. This task of judgment raises questions about not only individual forms of life, but also *the* human life form that all people share and which inspires judgment on the following set of questions: Which path of life belongs to a person according to their life forms, and how can they stay on this path under changing conditions? In what does a person place their hope? What sustains a person and those who are with him or her in and after the decision?

Decisions are made in the logic of algorithmic decision-making within the context of a huge and shifting world of other decisions. Decision-making in the logic of healthcare professionals entails subsuming a specific case under a general diagnostic or therapeutic rule. In the logic of patients and their relatives, decisions need to be made within a life-world in which the regular course of life is interrupted by diseases or accidents, and their life forms need to be meaningfully continued under (sometimes radically) different conditions. The art of decision-making is to combine these three logics in such a way that their respective strengths are maximized and their weaknesses minimized.

Healthcare professionals, patients and relatives who make shared decisions with the help of AI systems are, thus, called to find answers to the question of whether and how the life forms of those who are affected, hold dear and or cannot be ignored can be pursued or need to be altered. Additionally, such decision-making-processes will often entail the question of who will eventually bear the consequences of the decision. Judgements that lead to such decisions need to be directed towards this shared world so that viable paths for all who are affected will come into view (Ulrich, 2022). The question whether a decision can be regarded as *good* as well as morally and legally *correct*, depends on this kind of worldliness, a closeness to life as a shared existence in the continuity of its forms and practices– a worldliness that depends on judgment and its articulation within decision-making processes. Decisions, therefore, need to include the capacity of judgment both as subsuming a particular phenomenon under a general rule and as competence to discern.

Finding and exploring the world as a common one remains a continuous task of judgments sensitive to life forms in any settings of decision-making, including those who do not involve AI systems. But it will be a major task for the technical development of AI-assisted decision-making to eventually help to contribute to the constitution, discovery and renewal of such a shared reality. In this reality, people can find decisions that enable them to take the next step that is right for them and help them to continue their lives in ways that accord to their life forms. The application of a model of ethical judgment sensitive to life forms to the interaction between user and AI system that adequately addresses their extended interaction remains not only a matter of machine learning but also of human learning and cooperation in decision-making processes. In order to find decisions that resonate with the lives of all who are affected, professionals in the healthcare system need to learn how to utilize the strengths of AI systems without losing due reference to life forms and moral principles alike.

### Implications for Ethical Guidance and Practice of AI Development and Use

The brief overview and critical discussion of ethical frameworks for medical AI has demonstrated the need to supplement principle-based approaches with further perspectives highlighting the impact of life-worlds and the construction of shared realities. Besides striving for ethical correctness in line with a certain set of principles, emphasis must be placed on stakeholders’ good life in its various forms and practices. Notably, this viewpoint currently cannot be expected from AI-driven decision support machines or AI systems themselves: despite becoming increasingly powerful, even the largest generative AI models do not have a biography, they do not share a life-world with other individuals and they are not embodied. And even if, in some future, that would be the case, their “AI life-world” still would be significantly different from the human and a direct comparison is not helpful. Therefore, the specific human perspectives and life-world need to be adequately considered to avoid over-reliance and misplaced trust in AI-driven machines. Moral dangers, therefore, arise from an often unnoticed anthropomorphization of machines (e.g. in language) as well as from deliberate attempts to mimic and influence human emotions (Véliz, 2023). Such intricate factors and effects should be carefully considered when developing ethical guidance for the development and use of AI systems.

Our plea towards overcoming the limited perspective of principled approaches and to further consider the role of ethical judgment which cannot be circumvented must not only be understood as an ethical-theoretical position but has concrete consequences for AI development and, finally, also for healthcare practice. If we accept that principles alone do not guarantee ethicality and the moral world is rather a joint construction of reality than a matter of mere control, this sheds new light on the various domains discussed previously. Regarding codices and normative frameworks, we need to become aware that the guiding potential of ethical principles should not be overestimated, but that many other procedures and competencies are needed to let principles become effective in practice. Ethical principles do not include the rules of their application in themselves, but can only help for good outcomes if they are accompanied by the experienced and– as far as possible– unbiased judgement of relevant actors, be it in industry or public AI development facilities or by healthcare professionals in clinical practice. As a practical consequence, the development of AI ethics policies in healthcare should be accompanied by strengthening the moral competencies of developers and end-users for identifying potential sources of “moral danger” that might hinder a proper interpretation of ethical principles in practice. In addition, the integration of ethical judgments sensitive to life forms must be part of professional training and a task of organizational development in healthcare institutions. Our approach also calls for collaborative modes of shared decision-making in team meetings and collegial counselling (Makarius et al., 2020). Investing in the moral sensitivity and moral judgment of developers as well as end-users makes allowance for the fact that principles need to be interpreted according to the respective context and stand in a continuous interaction with the life forms of all who are affected.

Accordingly, information technology developers should be supported in perceiving themselves as true ethical agents whose decisions make a difference to the moral quality of the products they deliver to healthcare institutions. A mere legalistic view of following the rules (be it the legal norms or the ethics guidance of the company or academic community) does not suffice as principles need to be adjusted to the respective context. Principles neither interpret themselves nor contain the rules of their application. Moral philosophy has shown, by reference to the late Wittgenstein (1958), that humans do not only obey but “follow” rules. The way to find out whether we are following a rule or moral principle correctly is via common practice (Holtzman & Leich, 2014; Kripke, 1982; Wittgenstein, 1958).

Developers, therefore, need to become aware of both the normative relevance of life forms and practices for decision-making in healthcare, and biases and undue influences that make the ethical judgment deviating from its main intention. Regarding healthcare professionals, the recognition of the impact of life-worlds and joint procedures of interpretation might help not only in conventional clinical decision-making but particularly when it comes to the use of AI-driven support. Putting the AI in its proper place as being an immensely powerful instrument but yet not a human with a life-worldly experience is an important precondition for future healthcare that does not reduce patients to data but succeeds in adequately addressing the *conditio humana*.
